# Treatment Discontinuation Prediction in Patients With Diabetes Using a Ranking Model: Machine Learning Model Development

**DOI:** 10.2196/37951

**Published:** 2022-09-23

**Authors:** Hisashi Kurasawa, Kayo Waki, Akihiro Chiba, Tomohisa Seki, Katsuyoshi Hayashi, Akinori Fujino, Tsuneyuki Haga, Takashi Noguchi, Kazuhiko Ohe

**Affiliations:** 1 Nippon Telegraph and Telephone Corporation Tokyo Japan; 2 Department of Healthcare Information Management The University of Tokyo Hospital Tokyo Japan; 3 NTT DOCOMO, INC Tokyo Japan; 4 NTT-AT IPS Corporation Kanagawa Japan; 5 National Center for Child Health and Development Tokyo Japan

**Keywords:** machine learning, machine-learned ranking model, treatment discontinuation, diabetes, prediction, electronic health record, EHR, big data, ranking, algorithm

## Abstract

**Background:**

Treatment discontinuation (TD) is one of the major prognostic issues in diabetes care, and several models have been proposed to predict a missed appointment that may lead to TD in patients with diabetes by using binary classification models for the early detection of TD and for providing intervention support for patients. However, as binary classification models output the probability of a missed appointment occurring within a predetermined period, they are limited in their ability to estimate the magnitude of TD risk in patients with inconsistent intervals between appointments, making it difficult to prioritize patients for whom intervention support should be provided.

**Objective:**

This study aimed to develop a machine-learned prediction model that can output a TD risk score defined by the length of time until TD and prioritize patients for intervention according to their TD risk.

**Methods:**

This model included patients with diagnostic codes indicative of diabetes at the University of Tokyo Hospital between September 3, 2012, and May 17, 2014. The model was internally validated with patients from the same hospital from May 18, 2014, to January 29, 2016. The data used in this study included 7551 patients who visited the hospital after January 1, 2004, and had diagnostic codes indicative of diabetes. In particular, data that were recorded in the electronic medical records between September 3, 2012, and January 29, 2016, were used. The main outcome was the TD of a patient, which was defined as missing a scheduled clinical appointment and having no hospital visits within 3 times the average number of days between the visits of the patient and within 60 days. The TD risk score was calculated by using the parameters derived from the machine-learned ranking model. The prediction capacity was evaluated by using test data with the C-index for the performance of ranking patients, area under the receiver operating characteristic curve, and area under the precision-recall curve for discrimination, in addition to a calibration plot.

**Results:**

The means (95% confidence limits) of the C-index, area under the receiver operating characteristic curve, and area under the precision-recall curve for the TD risk score were 0.749 (0.655, 0.823), 0.758 (0.649, 0.857), and 0.713 (0.554, 0.841), respectively. The observed and predicted probabilities were correlated with the calibration plots.

**Conclusions:**

A TD risk score was developed for patients with diabetes by combining a machine-learned method with electronic medical records. The score calculation can be integrated into medical records to identify patients at high risk of TD, which would be useful in supporting diabetes care and preventing TD.

## Introduction

### Background

Diabetes is a chronic disease requiring both self-management and long-term management. Poor glycemic control increases the risk of complications, including cardiovascular and cerebrovascular diseases as well as macrovascular and microvascular diseases, such as nephropathy, retinopathy, and neuropathy [1-4]. To prevent the progression of these complications, adherence to dietary, exercise, and medication regimens is necessary [5]. Nonadherence has been shown to increase the risk of morbidity [4] and all-cause mortality [6].

Treatment discontinuation (TD), defined as dropping out of regular medical care, is likely to result in the worsening of glycemic control and progression of complications [3,4]. TD rates in patients with diabetes are rather high, ranging from 4% to 19% in the United Kingdom [3,4], 12% to 50% in the United States [7,8], and 13.5% to 56.9% in Japan [9,10]. Furthermore, patients who have previously discontinued treatment have been shown to have a 3-fold higher risk of repeated TD than those who have never done so [11].

### Prior Work

Preventing TD is crucial in the management of diabetes, and several studies have statistically analyzed the factors associated with TD [6-8,12]. Previously identified factors include younger age [6,13], smoking [6,14], poor glycemic control [6,13,15,16], high blood pressure [13], obesity [9], medications [12,16], employment status [8,17], region [18], transportation barriers [7,19,20], clinical appointments [20], and complications [21]. The most commonly used statistical hypothesis tests are *t* test and chi-square test. However, a review [22] pointed out a variety of multilevel factors in association with TD with inconsistent findings. It has remained difficult for clinicians to carefully discern each patient’s risk of TD.

Machine learning (ML) may be useful for predicting each patient’s risk of TD by taking into account a wide variety of factors. Statistics focus on *explaining outcomes with data*, whereas ML focuses on *predicting outcomes with data* [23]. Although ML cannot identify consistent factors, it can inform clinicians about who is a high-risk patient for TD. It could help clinicians shift their time spent on identifying high-risk patients to encouraging them to continue treatment. According to a systematic review by Carreras-García et al [24], most studies designed their model as a binary classification problem [25] that classified scheduled appointments based on whether they were kept or missed. Furthermore, the most commonly used model was logistic regression, and the most frequently used metric was the area under the receiver operating characteristic curve (AUROC). However, as a binary classification outputs the probability of a missed appointment (MA) occurring after a predetermined period, it is limited in its ability to estimate the magnitude of TD risk in patients with inconsistent intervals between appointments. Even if a patient missed an appointment, if the frequency of visits was maintained such that their condition did not worsen thereafter, the TD risk of the patient would be low. An MA is a necessary but not sufficient condition for TD.

### Goal of This Study

In this study, we aimed to develop a novel method of calculating TD risk via ML. We designed a prediction model of TD as a ranking problem with imbalanced data to compare patients by length of time until TD. The ranking problem [26] is an application of survival time analysis [27]. Cox regression [28] is generally used in statistical analysis, whereas the ranking model is used in ML [29-31]. Cox regression is a model of the hazard function in which the effects of the explanatory variables on outcomes are predetermined, requiring an assumption that they remain constant over time [28]. In contrast, the ranking model does not require this assumption and makes flexible use of the variables. Furthermore, because there was a concern that the learning model would have a heavier bias toward TD cases than treatment continuation (TC) cases, the sampling was devised on the basis of the findings of the imbalanced data.

The contributions of this work are as follows:

1. This study designed a prediction model of TD as a ranking problem with imbalanced data, which allows for a comparison of patients’ risk of TD with the time remaining before TD. This is the first study to use a machine-learned ranking model to predict TD.
2. The mean (95% confidence limits) of the C-index for the TD risk score obtained with the model was 0.749 (0.655, 0.823). This was higher than 0.662 (0.574, 0.748), which was obtained with the Cox regression model; the results for the AUROC and area under the precision-recall curve (AUPRC) were similar.

## Methods

### Ethics Approval

This study was approved by the research ethics committees of the Graduate School of Medicine and Faculty of Medicine at the University of Tokyo (approval number: 10705) and was conducted in accordance with the Declaration of Helsinki. Informed consent was obtained, and an opportunity to opt out of participation was provided.

### Study Population

All data were collected from electronic health records (EHRs) at the University of Tokyo Hospital, which included 7551 patients who visited the hospital after January 1, 2004, and had diagnostic codes indicative of diabetes. Characteristics of patient in the training and test data are shown in Table 1.

**Table 1:** Characteristics of patients in the training and test data.

Characteristics			Training data (n=6509)				Test data (n=1042)		
Group			TD^a^ (n=204, 3.13%)		TC^b^ (n=6305, 96.86%)		TD (n=38, 3.65%)		TC (n=1004, 96.35%)
Number of appointments, mean (SD)			4.8 (3.3)		10.4 (5.0)		3.1 (2.6)		5.8 (4.1)
Number of missed appointments, mean (SD)			1.6 (1.2)		1.6 (1.2)		1.2 (0.5)		1.3 (0.7)
**Age (years), mean (SD)**			62.6 (15.9)		66.0 (12.6)		59.9 (15.0)		61.1 (14.1)
	<20, n (%)	0 (0)		3 (0.05)		0 (0)		1 (0.10)	
	20-30, n (%)	5 (2.50)		45 (0.71)		1 (3)		25 (2.49)	
	30-40, n (%)	14 (6.90)		204 (3.24)		4 (11)		63 (6.27)	
	40-50, n (%)	28 (13.70)		452 (7.17)		6 (16)		117 (11.65)	
	50-60, n (%)	31 (15.20)		883 (14)		6 (16)		188 (18.73)	
	60-70, n (%)	47 (23)		1950 (30.93)		8 (21)		310 (30.88)	
	≥70, n (%)	79 (38.70)		2768 (43.90)		13 (34)		300 (29.88)	
**Sex, n (%)**									
	Male	127 (63.30)		3777 (59.90)		25 (66)		594 (59.16)	
	Female	77 (37.70)		2528 (40.10)		13 (34)		410 (40.84)	
**Hospital visit interval in days, mean (SD)**			65.9 (33.1)		57.3 (23.9)		56.2 (65.5)		49.0 (21.0)
	<30, n (%)	4 (2)		283 (4.49)		7 (18)		127 (12.65)	
	30-60, n (%)	72 (35.30)		3237 (51.34)		15 (39)		511 (50.90)	
	60-90, n (%)	66 (32.30)		2140 (33.94)		3 (8)		177 (17.63)	
	≥90, n (%)	26 (12.80)		415 (6.58)		2 (5)		39 (3.88)	
First visit, n (%)			36 (17.70)		230 (3.65)		11 (29)		150 (14.94)
**HbA_1c_^c^ (NGSP^d^),%, mean (SD)**			7.1 (1.2)		7.0 (1.0)		7.0 (1.1)		7.0 (1.1)
	<6, n (%)	31 (15.20)		770 (12.21)		6 (16)		118 (11.75)	
	6-7, n (%)	64 (31.40)		2281 (36.18)		12 (32)		382 (38.05)	
	7-8, n (%)	48 (23.50)		1788 (28.36)		9 (24)		285 (28.39)	
	≥8, n (%)	33 (16.20)		632 (10.02)		4 (11)		148 (14.74)	
	Missing value, n (%)	28 (13.70)		834 (13.23)		7 (18)		71 (7.07)	
**TG^e^, mg/dL, mean (SD)**			182.2 (167.4)		143.5 (96.5)		199.0 (239.1)		160.5 (120.9)
	<30, n (%)	0 (0)		4 (0.06)		0 (0)		0 (0)	
	30-150, n (%)	91 (44.60)		3601 (57.11)		15 (39)		550 (54.78)	
	150-300, n (%)	65 (31.90)		1631 (25.87)		10 (26)		291 (28.98)	
	300-750, n (%)	16 (7.80)		213 (3.38)		3 (8)		72 (7.17)	
	≥750, n (%)	3 (1.50)		11 (0.17)		1 (3)		6 (0.60)	
	Missing value, n (%)	29 (14.20)		845 (13.40)		9 (24)		85 (8.47)	
**HDL^f^, mg/dL, mean (SD)**			58.6 (15)		60.6 (16.9)		54.4 (20.3)		56.6 (16.8)
	<20, n (%)	0 (0)		2 (0.03)		0 (0)		0 (0)	
	20 to <40, n (%)	15 (7.40)		387 (6.14)		8 (21)		130 (12.95)	
	40 to <100, n (%)	159 (77.90)		4882 (77.43)		20 (52)		759 (75.60)	
	≥100, n (%)	3 (1.50)		126 (2)		1 (3)		15 (1.49)	
	Missing value, n (%)	27 (13.20)		908 (14.40)		9 (24)		100 (9.96)	
**LDL^g^, mg/dL, mean (SD)**			121.6 (31.3)		111.6 (26.8)		119.9 (33.7)		113.0 (35.0)
	<60, n (%)	2 (1)		107 (1.70)		1 (3)		26 (2.59)	
	60-120, n (%)	64 (31.40)		2700 (42.82)		7 (18)		338 (33.67)	
	120-140, n (%)	36 (17.70)		988 (15.67)		2 (5)		125 (12.45)	
	≥140, n (%)	32 (15.70)		532 (8.44)		5 (13)		120 (11.95)	
	Missing value, n (%)	70 (34.30)		1978 (31.37)		23 (61)		395 (39.34)	
**TCho^h^, mg/dL, mean (SD)**			201.6 (44.5)		189.5 (32.8)		193.3 (36.6)		192.9 (43.4)
	<130, n (%)	2 (1)		152 (2.41)		1 (3)		50 (4.98)	
	130-220, n (%)	111 (54.40)		4202 (66.65)		20 (53)		650 (64.74)	
	220-240, n (%)	23 (11.30)		516 (8.18)		6 (16)		97 (9.66)	
	240-280, n (%)	15 (7.40)		246 (3.90)		1 (3)		77 (7.67)	
	≥280, n (%)	5 (2.50)		43 (0.68)		0 (0)		29 (2.89)	
	Missing value, n (%)	48 (23.50)		1146 (18.18)		10 (26)		101 (10.06)	

^a^TD: treatment discontinuation.

^b^TC: treatment continuation.

^c^HbA_1c_: hemoglobin A_1c_.

^d^NGSP: National Glycohemoglobin Standardization Program.

^e^TG: triglyceride.

^f^HDL: high-density lipoprotein.

^g^LDL: low-density lipoprotein.

^h^TCho: total choline.

**Figure 1:**
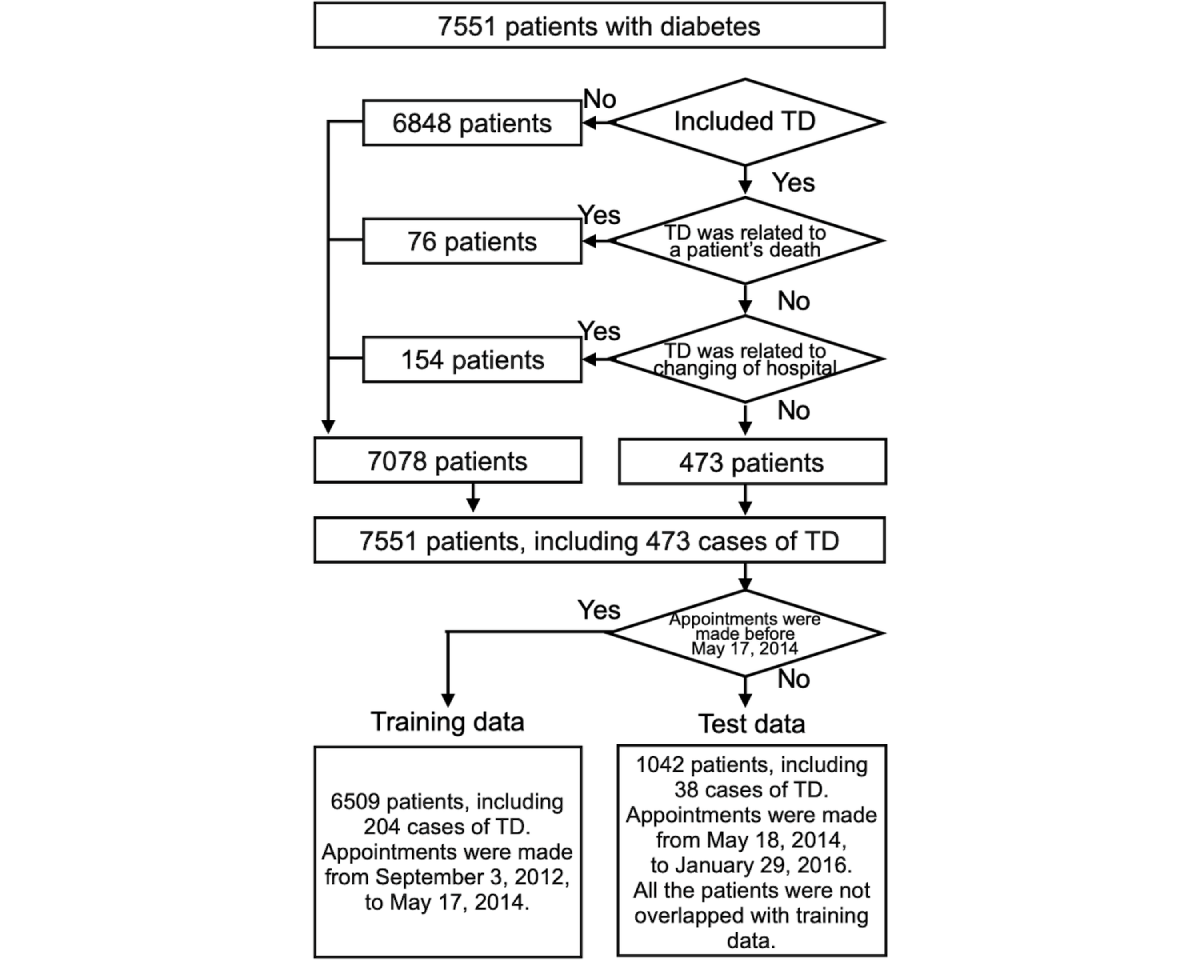
Illustration of patient selection and data preprocessing. TD: treatment discontinuation.

The data were recorded in the EHRs between September 3, 2012, and January 29, 2016. As illustrated in Figure 1, based on the calendar date, two-thirds of the data (days: 828/1243, 66.6%) were used for training (between September 3, 2012, and May 17, 2014) and the remaining one-third (days: 415/1243, 33.4%) was used for testing (between May 18, 2014, and January 29, 2016). The records used for training were not used for testing to ensure that the same patients were not included in both groups. A total of 6509 patients (204 cases of TD) were included in the training group, and 1042 patients (38 cases of TD) were included in the testing group.

### Definition of TD

The TD of a patient was defined as missing a scheduled clinical appointment and having no hospital visits within 3 times the average number of days between the visits of the patient and within 60 days. Each patient’s average number of days between visits was calculated from the last 3 visit days. In other words, if 3 times the average number of days between visits was greater than 60 days, then 60 days was used as the threshold. Otherwise, 3 times the average number of days between visits was used as the threshold.

Other studies have defined TD as the lack of hospital visits over a particular threshold of time (between 1 day and 6 months) [6-8,12-21]. When the threshold was set at 60 days, 336 cases of TD were detected in the training data and 65 cases of TD were detected in the test data, but there was a trend that patients with longer visit intervals were more likely to be judged as TD cases. It is not easy to set appropriate thresholds for outpatients whose hospital visits are at inconsistent intervals. Next, when the threshold was set to 3 times the average number of days between visits, 218 cases of TD were detected in the training data and 54 cases of TD were detected in the test data, but patients with shorter visit intervals tended to be more likely to be judged as TD cases or judged as having a risk of TD. Therefore, we included both conditions in the definition.

To ensure accurate TD detection, a physician, one of the coauthors, verified that the above definition was met and excluded cases of patient death or changes in care setting.

### Length of Treatment Until Discontinuation

Length of treatment was measured in 2 ways. First, TD (*p_m_, t*_m_) was defined as the number of days from the date *t_m_* to the missed scheduled clinical appointment associated with TD for the patient *p_m_* who had TD (or possible TD). In the second way, TC (*p_n_, t_n_*) was defined as the number of days from the date *t_n_* to the most recently recorded visit for the patient *p_n_* who had no TD.

**Figure 2:**
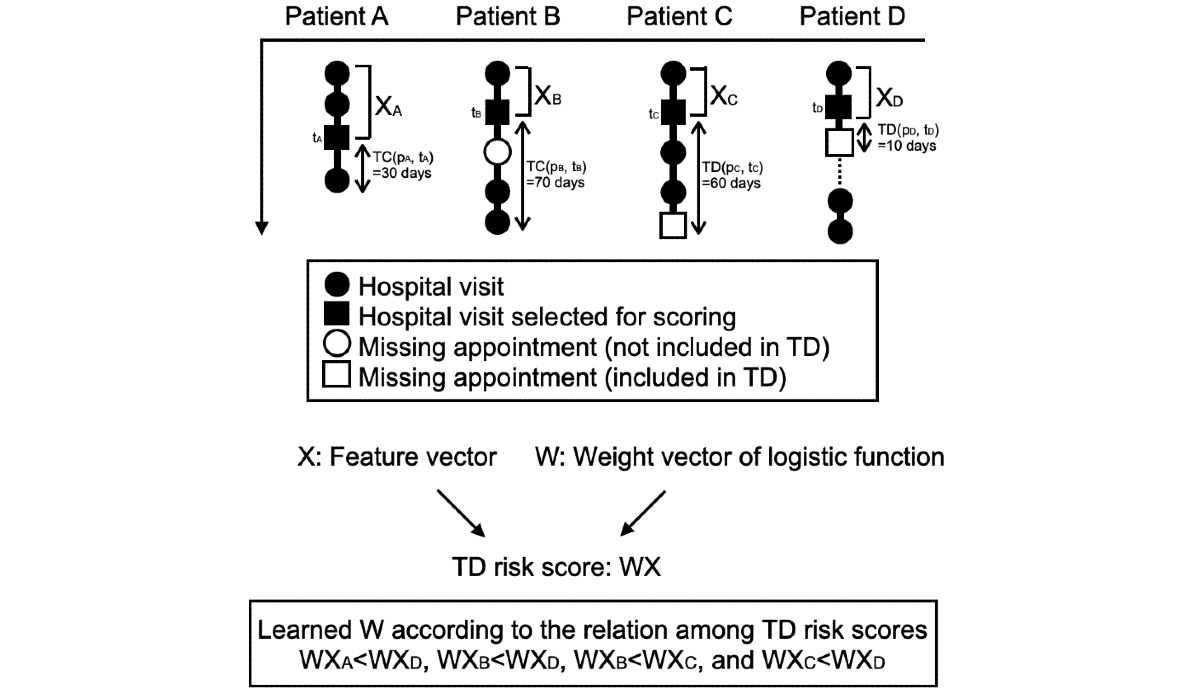
Examples of the value of the treatment discontinuation (TD) risk. TC: treatment continuation; W: weight vector; X: feature vector.

For example, as shown in Figure 2, in the case of patient A, there were 30 days from *t_A_* to the most recently recorded visit, so TC (*p_A_, t_A_*) was set to 30 days. In the case of patient C, there were 60 days from *t_C_* to the missed scheduled clinical appointment associated with TD, so TD (*p_C_, t_C_*) was set to 60 days.

### Class Design

The classification *y_m,n_* was based on the difference between a pair of treatment lengths. Here, *y_m,n_*=+1 for the pair of TD (*p_m_, t_m_*) for the patient *p_m_* and the date *t_m_* and TD (*p_n_, t_n_*) for the patient *p_n_* and the date *t_n_* if TD (*p_m_, t_m_*) is shorter than TD (*p_n_, t_n_)* and the pair of TD (*p_m_, t_m_*) and TC (*p_n_, t_n_*) if TD (*p_m_, t_m_*) is shorter than TC (*p_n_, t_n_*). *y_m,n_*=–1 for the pair of TD (*p_m_, t_m_*) and TD (*p_n_, t_n_*) if TD (*p_m_, t_m_*) is longer than TD (*p_n_, t_n_*) and for the pair of TC (*p_m_, t_m_*) and TD (*p_n_, t_n_*) if TC (*p_m_, t_m_*) is longer than TD (*p_n_, t_n_*).

The classification was performed only when the patients had different times until TD, or when one patient had TD and the other had TC, where TC (*p_n_, t_n_*) was longer than TD (*p_m_, t_m_*). The classification was not performed on other occasions because the difference in time until TD between the 2 patients could not be compared. For the examples shown in Figure 2, the classes of the pair of TC (*p_A_, t_A_*) and TD (*p_D_, t_D_*) and that of TC (*p_B_, t_B_*) and TD (*p_D_, t_D_*), TC (*p_B_, t_B_*) and TD (*p_C_, t_C_*), and TD (*p_C_, t_C_*) and TD (*p_D_, t_D_*) were all set to −1.

### Feature Design

To ensure that the factors related to TD were included, we designed a feature vector *x_n_* for patient *p_n_* at time *t_n_*, representing the clinical conditions beginning with the initial visit and lasting until just before *t_n_*. In total, 149,699 features, 51,778 qualitative features and 97,921 quantitative features, were used. Table 2 describes the features used for the prediction.

We designed the features using 3 classes of representation. The first included detailed demographic and clinical conditions (sex, age, previously consulted medical departments, diagnosed diseases, and prescribed medications). These had numerous features, most of which had a 0 value, leading to a very sparse representation.

The second class included changes occurring during the treatment of a patient to identify the risk of TD at each hospital visit. For example, we used the accumulated number of hospital visits, length of prescription time, number of medications prescribed, laboratory results, day of the week an appointment was scheduled, the interval between the date on which a clinical appointment was made and the scheduled appointment date, and the weather conditions on the appointment day. Detailed histories of hospital visits were included because features related to when and how appointments were made influenced the accuracy of the predicted MAs in our previous work [25].

The third class included data from public databases beyond the EHR. For instance, to represent the distance from a patient’s home to the hospital, we used a geographic information system and measured the distance and travel time. We also used information regarding patient occupations. The observed values of each quantitative variable, for example, blood test results, were linearly transformed (normalized) to make the variance of each variable equal to 1. The transformed variable was then assigned to the vector.

**Table 2:** Description of explanatory variables used for prediction.

Primary and secondary categories		Qualitative variables (n=51,778), n (%)	Quantitative variables (n=97,921), n (%)	Characteristic feature (reference)
**Attribute**				
	Sex and age	4 (0.01)	5 (0.01)	Sex and age
	Address	492 (0.95)	492 (0.50)	Distance and time duration from the house to the hospital by public transport (geographic information system)
	Insurance	67 (0.13)	3 (0)	Business-type category (health insurance societies of companies)
**Consultation**				
	Medical department, outpatient, and inpatient	267 (0.52)	514 (0.52)	Previously and recently consulted medical departments
	Subject	8021 (15.49)	13,108 (13.39)	Subject categories of consultation assigned by each medical department
	Time	33 (0.06)	105 (0.11)	Late arrival for an appointment
	Appointment (intervals and changes)	74 (0.14)	197 (0.20)	Interval between the date on which a clinical appointment was made and scheduled appointment date
**Medicine**				
	Directions of each medicine	10,346 (19.98)	17,678 (18.05)	How many times a day medication is taken
	Doses of each medicine	4570 (8.83)	33,403 (34.11)	Total amount of medication per day
	Component	2332 (4.50)	5082 (5.19)	Component (medicine code defined by the Ministry of Health, Labor and Welfare)
	Medical department, outpatient, and inpatient	324 (0.63)	678 (0.69)	Medication for outpatient to the department of Diabetes and Metabolic Diseases
	Disease (recovered from and under treatment)	21,977 (42.44)	22,012 (22.48)	Disease category under care and recovered (ICD-10^a^)
**Laboratory tests**				
	Medical department, outpatient, and inpatient	170 (0.33)	357 (0.36)	HbA_1c_^b^, HDL-C^c^, LDL-C^d^, TG^e^, TCho^f^, etc
	Order, exam and intervals	219 (0.42)	462 (0.47)	Interval between tests
	Results	297 (0.57)	658 (0.67)	Categorized result according to the criteria (Diabetes Medical Guideline)
	Physiological tests (order, exam, and intervals)	2237 (4.32)	2801 (2.86)	Interval between tests
	Surgery (procedure)	336 (0.65)	338 (0.35)	Procedure name
	Nutritional guidance (medical department, outpatient, and inpatient)	12 (0.05)	28 (0.03)	Guidance for inpatient to the department of Diabetes and Metabolic Diseases

^a^ICD-10: International Classification of Diseases, Tenth Revision.

^b^HbA_1c_: hemoglobin A_1c_.

^c^HDL-C: high-density lipoprotein.

^d^LDL-C: low-density lipoprotein.

^e^TG: triglycerides.

^f^TCho: total choline.

All the features were generated by processing variables obtained from the EHRs. The category with the highest number of variables was medicine. Raw categorical variables such as medicine name, component, units, inpatient and outpatient category, and department that prescribed the medicine were extracted. Raw numerical variables such as amount, dosage, and number of days or times were extracted. In addition, new numerical variables were generated by combining categorical and numeric variables such as pairs of medicine name and amount, pairs of medicine name and dosage, and pairs of medicine name and number of days or times. New categorical variables such as pairs of medicine name and inpatient and outpatient category and pairs of medicine name and department were also generated. The category with the second highest number of features was disease. Raw categorical variables such as disease name; disease category defined by International Classification of Diseases, Tenth Revision; treatment status (under treatment and recovering); and disease type (primary disease and secondary disease) were extracted. In addition, new categorical variables such as pairs of disease name and treatment status and pairs of disease name and disease type were generated. New numerical variables were also generated by counting the number of diseases that were under treatment and recovered for each disease category. The variables of the other categories were as follows. From the attribute category, categorical variables such as sex, names of regions and cities, insurance categories, and business-type categories were extracted. Numerical variables such as age and copayment rates were extracted. Distance and travel time were generated as new numerical variables using geographic information system from region and city names, as described in the third representation class. From the consultation category, categorical variables such as department, inpatient and outpatient category, and subject name of the reservation slot were extracted. Numerical variables such as time of arrival, appointment, clinic start, and clinic end were extracted. These time intervals were generated as new numerical variables. From the appointment category, categorical variables such as department and appointment status (new, change, and cancellation) were extracted. Numerical variables such as time of registration and reservation were extracted. The new numerical variables were generated, as described in the second representation class. From the laboratory and physiological tests categories, categorical variables such as test name, department, and inpatient and outpatient category were extracted. Numerical variables such as test values were extracted. From the surgery category, categorical variables such as operative name were extracted. From the nutritional guidance category, categorical variables such as department and inpatient and outpatient categories were extracted.

Most features were generated using the following 3-step procedure. First, raw variables were extracted from each category, tied to their recorded times, and classified into categorical variables (eg, names of diagnosed diseases) and numeric variables (eg, number of medicines prescribed). Second, Categorical variables were further classified into raw categorical variables and frequency-transformed categorical variables. Third, the combinations of the raw categorical variables and the statistics of the frequency-transformed categorical variables were computed with varying window sizes to generate qualitative features and quantitative features, respectively. Numeric variables were transformed to linear and logarithmic scales, and their statistics were computed with varying window sizes to generate quantitative features. 4 statistics were used for feature generation: minimum, maximum, mean, and SD. To relate the most recent trends in circumstances to the TD risk score, periods of 3 months, 6 months, and 1 year before the target time were used as window sizes. A categorical variable was also added to indicate missing data if a feature was present for a shorter time than the window size.

For example, from the attribute category, the features sex, age, address, and insurance were extracted to express demographic conditions. The features of sex consisted of 1 qualitative variable representing male or female, 3 quantitative variables representing its frequencies with the 3 window sizes, and 3 qualitative variables representing their missing values. The frequencies of the sex variable itself have no meaning, but because it is a variable that is always listed in each EHR, it was used to represent the number of EHRs in the window size. The features of age consisted of 2 quantitative variables of linear and logarithmic scales. The features of address consisted of 48 quantitative variables of the 4 statistics of the 2 scales of the distance and travel time from a patient’s home to the hospital with 3 window sizes, 48 qualitative variables representing their missing values, and 444 quantitative and qualitative variables representing the names of regions and cities and their frequencies. The features of insurance consisted of 67 qualitative variables representing insurance categories and business-type categories and 3 quantitative variables representing copayment rates.

### Model Design

We established a TD risk prediction method based on the parameters of the machine-learned ranking model. There are several objective function designs for ranking models [32,33]. In particular, pointwise [26], pairwise [34-36], and listwise [37,38] approaches have been proposed. Furthermore, several learning algorithms have been developed, including ones that use logistic regression, neural networks [39], and boosting [40].

We designed the model on the basis of the pairwise approach and used logistic regression. The pairwise approach was appropriate as the only rating scale for learning was the TD risk score. Logistic regression was selected because it was the most frequently used approach in related work [24] and because it was used in our previous work [25].

We hypothesized that the risk of TD of patient *p_m_* can be calculated from a feature vector *x_m_* that incorporates a variety of patient information up to time *t_m_*. Therefore, we assumed that the scalar TD risk can be represented by the inner product of a weight vector and the feature vector, that is, *w* ⋅ *x_m_*. To obtain the weight vector *w*, we modeled the probability that patient *p_m_* at time *t_m_* would discontinue treatment earlier than *p_n_* at *t_n_*, with *x_m_* and *x_n_* attributed to *y_m,n_* with the logistic regression:

*P* (*y_m_*_,_*_n_* | *x_m_*,*x_n_*;*w*) = 1 / {1 + exp [– *y_m_*_,_*_n_w* (*x_m_* – *x_n_*)]}

The notation *w* (*x_m_-x_n_*) denotes the scalar product of *w* and *x_m_-x_n_*.

### ML Design

The ranking method, based on the pairwise approach, requires pairs of data for optimizing the parameters of the model. In general, *n*(*n*-1)/2 pairs can be generated for *n* records with no censoring. As this study included censored data that were TCs, all pairs for optimization must satisfy the abovementioned combination rule. There was also a concern that the model would have a heavier bias toward TD cases than toward TC cases. According to survey papers [41-43] on biased data, sampling has often been attempted as a way to solve this problem [44,45]. We took the means of sampling 1 record from each patient to prevent biased learning on a small number of patients. When the *w* estimate was computed, we randomly selected 1 recorded date of a hospital visit for each patient and used the date *t_m_* or *t_n_* as the starting point of TD or TC to calculate TD (*p_m_, t_m_*) or TC (*p_n_, t_n_*). The number of all pairs satisfying the abovementioned combination rule with the sampling was 867,574 in the training data and 17,038 in the test data. The computational complexity of pairwise-based ranking learning is O(n^2^). The sampling results in a slightly reduced computational cost.

When the training data size, *N*, is smaller than the dimension of the feature vectors, or when sampling of the training data is biased, a maximum-likelihood estimation often overfits a logistic regression model to the training data, leading the model to rank many new patients inaccurately. We used an L2-norm regularization method [23] to mitigate overfitting and improve the generalizability of the model, as we did in our previous study [25].

Using training data [(*x*_1_, *x*_2_, *y*_1,2_),..., (*x*_1_, *x*_N_, *y*_1, N_),..., (*x*_2_, *x*_3_, *y*_2,3_),..., (*x_m_*, *x_n_*, *y_m_*_,_*_n_*),..., (*x*_N–1_, *x*_N_, *y*_N–1,N_)], we estimated *w* as follows:



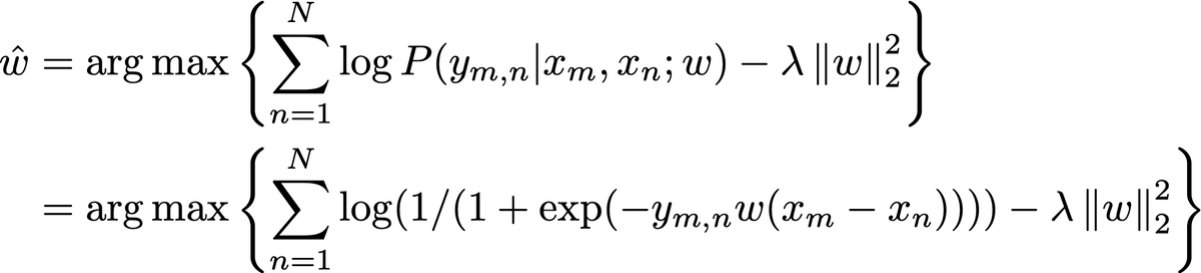



where the squared L2-norm of *w*, 
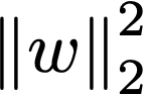
, is an L2-norm regularizer that acts as a mitigating penalty to provide large absolute weight values only to frequently occurring features in the training data.

The symbol *λ* is a hyperparameter for regularization and was tuned as follows: the training data were randomly split into 2 sets of data and used in a 2-fold cross-validation test; for each test, the prediction accuracy was evaluated with one set of data for training and the other set of data for testing, with *λ* set to 0.1, 0.2, 0.5, 1, 2, 5, 10, 20, 50, and 100. The value of *λ* at which the average prediction accuracy of the 2 tests was highest was chosen.

### TD Risk Score Design

The TD risk score of patient *p_m_* at time *t_m_* is represented by the logit value *w* ⋅ *x_m_*. The higher the value of the TD risk, the earlier TD is predicted to occur. Figure 2 shows an example of the TD risk value.

### Statistical Analysis

We implemented the model and ML optimization in-house in C and Python 3.7 and used it in all the experiments.

## Results

### Distribution of TD and TC

**Figure 3:**
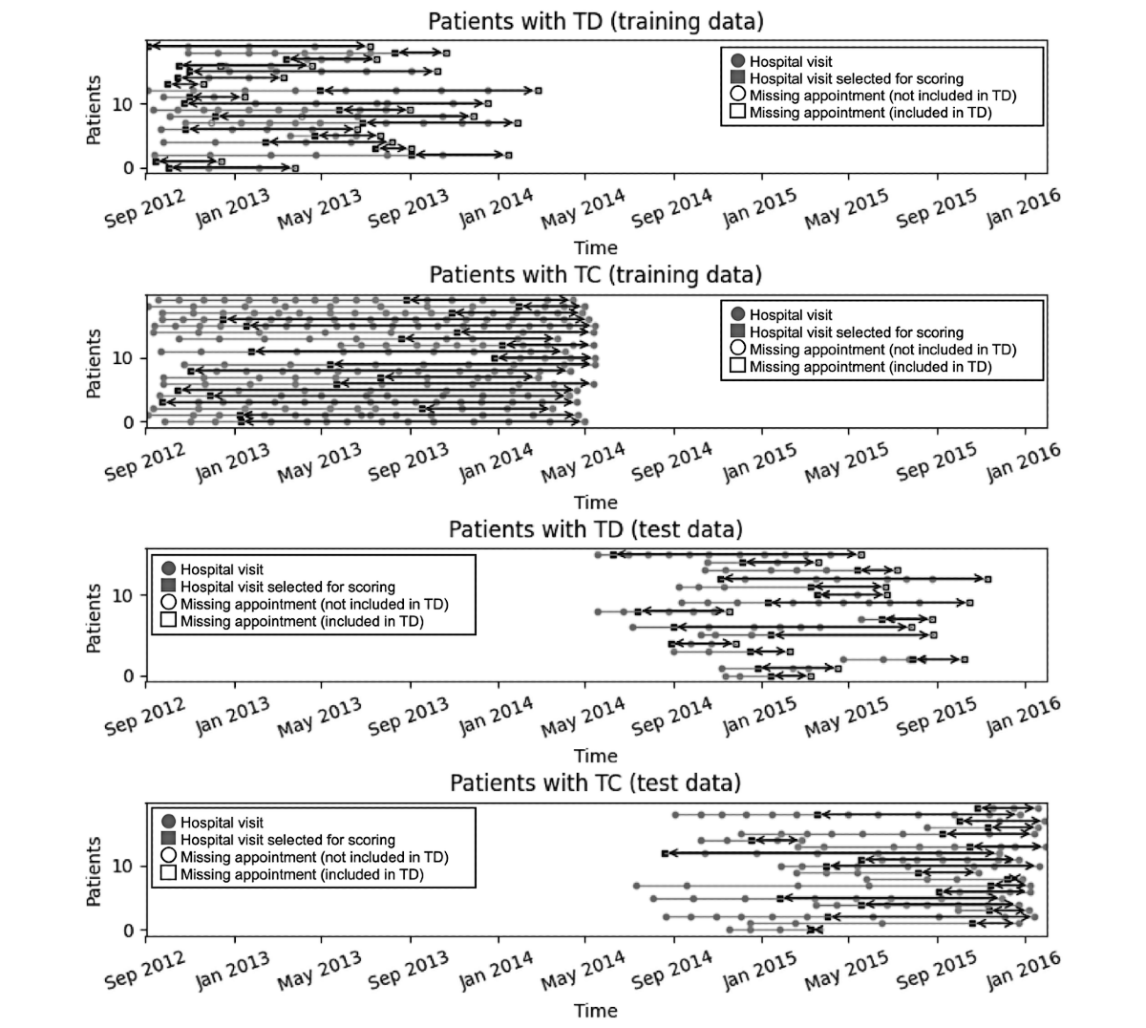
Example of distribution of visit and appointment dates. TC: treatment continuation; TD: treatment discontinuation.

The detailed demographic data are shown in Table 1. The average numbers of appointments by patients with TD and TC were 4.8 and 10.4, respectively, in the training group and 3.1 and 5.8, respectively, in the testing group. The difference in distribution was because of the training and test data were classified according to whether or not they had a history of hospital visits before May 17, 2014, and the duration of the training data (828 days) was approximately twice that of the test data (415 days). Furthermore, as shown in Figure 3, the training data included patients who had been attending the hospital since before September 3, 2012, which was the starting point for the experiment; thus, patients with TC in the training data tended to have more appointments. In contrast, patients with TC in the test data tended to have fewer appointments, as these data were limited to patients who had attended the hospital since May 17, 2014. However, the number of appointments for patients with TD was low for both training and test data as patients with TD generally had shorter hospital visits. The average numbers of MAs by patients with TD and TC were 1.6 and 1.6, respectively, in the training group and 1.2 and 1.3, respectively, in the testing group.

### Predictive Performance Against TD

**Figure 4:**
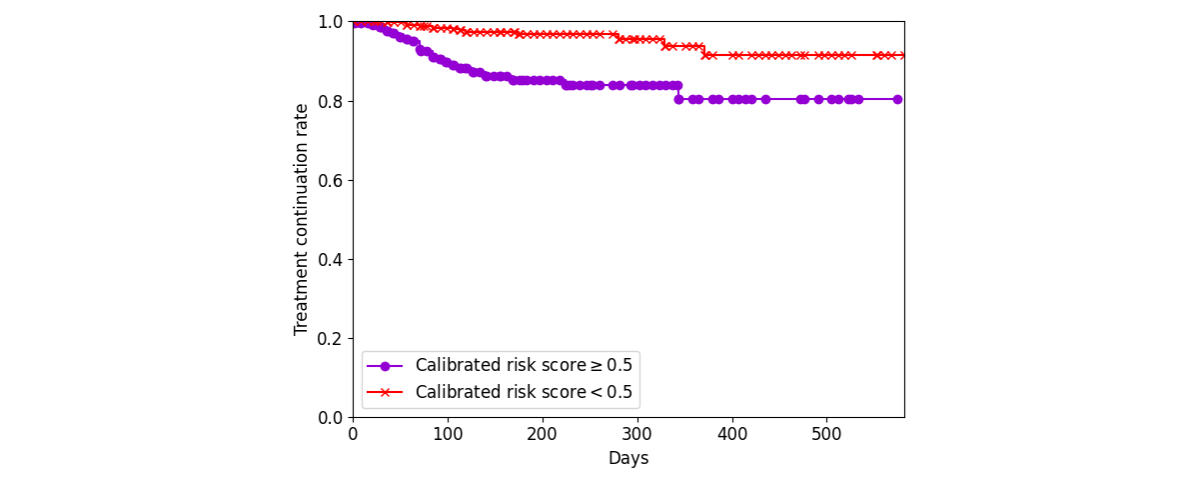
Kaplan-Meier curves displaying the probability of treatment discontinuation (TD) for the 2 groups of test data divided by the median TD risk scores obtained from the training data.

The hyperparameter *λ* of the machine-learned ranking model was tuned with 2 cross-validations, and it was set to 10 in the testing stage. The C-index of the predicted ranking was calculated as the number of correctly ranked pairs divided by the total number of comparable pairs. During testing, the TD risk score generated by the algorithm performed well, with a C-index (95% confidence limits) of 0.749 (0.655, 0.823), and outperformed the Cox regression model, with a C-index (95% confidence limits) of 0.662 (0.574, 0.748). As shown by the Kaplan-Meier curve in Figure 4, it was able to correctly model the population at high risk for TD. 10.3% (36/349) of the patients whose calibrated risk scores were ≥0.5 discontinued treatment within 100 days, whereas 93.9% (651/693) of the patients whose scores were <0.5 continued treatment for over 1 year.

The number of TD cases was much smaller in the data used in this study than the number of patients who did not interrupt their visits. As validation with the C-index alone might not be sufficient to evaluate the performance in the case of imbalanced data [45,46], the AUPRC was used in addition to the AUROC to evaluate whether the risk score could predict TD in a specific period, as shown in Table 3. Both the AUROC and AUPRC of the TD risk score were higher than those of the Cox regression model.

TD prediction within 6 months showed an AUROC (95% confidence limits) of 0.741 (0.641, 0.833) and an AUPRC (95% confidence limits) of 0.335 (0.193, 0.499). These values at 1 year were 0.758 (0.649, 0.857) and 0.713 (0.554, 0.841), respectively.

**Figure 5:**
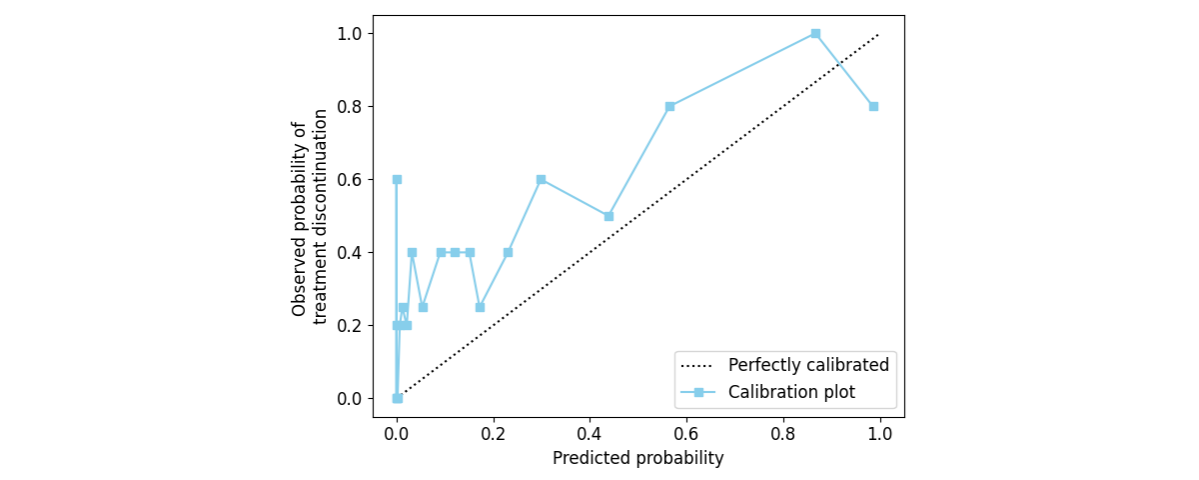
The distribution of the predicted probability and observed probability of treatment discontinuation is shown in a line chart. Each point represents the observed and predicted probabilities for each of the 20 segments of the test population.

Subsequently, the TD risk score was converted to a range of 0 to 1 to validate the performance of risk stratification. As shown in the calibration plot using the test data in Figure 5, the observed and predicted TD rates were relatively correlated. These results indicate that the TD risk score can provide clinicians with information about the risk of TD in advance with favorable predictive performance and improve patient outcomes by providing room for interventions to avoid interruptions.

**Table 3:** Predictive performance against TD^a^.

Months	AUROC^b^, mean (95% confidence limits)		AUPRC^c^, mean (95% confidence limits)	
	Ranking model	Cox model	Ranking model	Cox model
2	0.747 (0.607, 0.868)	0.668 (0.544, 0.787)	0.081 (0.024, 0.299)	0.035 (0.016, 0.071)
3	0.776 (0.666, 0.870)	0.691 (0.581, 0.793)	0.228 (0.090, 0.412)	0.136 (0.052, 0.262)
4	0.748 (0.637, 0.844)	0.641 (0.531, 0.746)	0.290 (0.139, 0.470)	0.156 (0.072, 0.278)
5	0.751 (0.651, 0.843)	0.666 (0.557, 0.768)	0.309 (0.163, 0.483)	0.215 (0.107, 0.360)
6	0.741 (0.641, 0.833)	0.645 (0.533, 0.751)	0.335 (0.193, 0.499)	0.236 (0.127, 0.379)
7	0.746 (0.645, 0.841)	0.660 (0.547, 0.764)	0.414 (0.254, 0.576)	0.308 (0.172, 0.468)
8	0.752 (0.650, 0.846)	0.677 (0.565, 0.781)	0.478 (0.311, 0.635)	0.384 (0.227, 0.544)
9	0.756 (0.654, 0.850)	0.675 (0.561, 0.785)	0.510 (0.337, 0.670)	0.438 (0.269, 0.601)
10	0.750 (0.646, 0.846)	0.691 (0.569, 0.800)	0.570 (0.402, 0.726)	0.562 (0.389, 0.708)
11	0.732 (0.625, 0.830)	0.680 (0.561, 0.793)	0.609 (0.442, 0.757)	0.597 (0.426, 0.742)
12	0.758 (0.649, 0.857)	0.687 (0.569, 0.798)	0.713 (0.554, 0.841)	0.645 (0.485, 0.784)

^a^TD: treatment discontinuation.

^b^AUROC: area under the receiver operating characteristic curve.

^c^AUPRC: area under the precision-recall curve.

### Items With the Largest Coefficient Values

The items with the largest coefficient values were examined to check for leakage, wherein unintended information is used for prediction and degrades the performance of the model. The 5 highest and the 5 lowest items are shown in Table 4. The specific mechanism by which each item contributes to the prediction is difficult to discuss at this time, but there were no items among the top 5 that suggested obvious leakage.

**Table 4:** Top 5 and bottom 5 explanatory variables obtained from the training set.

Category	Weight size	Feature
Top 1	8.1	Frequency of visits with the reservation at the department of cardiovascular medicine within 3 months
Top 2	5.2	Frequency of visits with no letter of reference within 6 months
Top 3	5.2	Frequency of visits with no letter of reference within 3 months
Top 4	5.2	Frequency of visits with the reservation before an operation in the department of cardiovascular medicine
Top 5	5.2	Frequency of laboratory tests of protein in urine within 6 months
Bottom 1	−28	Frequency of blood pressure tests within 3 months
Bottom 2	−25	Frequency of appointments of carotid artery ultrasound examination within 3 months
Bottom 3	−16	Frequency of carotid echo tests within 3 months
Bottom 4	−15	Frequency of laboratory tests of HbA_1c_^a^ within 6 months
Bottom 5	−15	Frequency of laboratory tests of HbA_1c_ within 1 year

^a^HbA_1c_: hemoglobin A_1c_.

## Discussion

### Principal Findings

In this study, we generated a prediction model for the risk of TD using approximately 150,000 explanatory variables extracted from EHRs and advanced machine-learned techniques. The accuracy of the model’s prediction was validated.

### Comparison With Prior Work

ML has been used in almost all aspects of diabetic research, especially in biomarker identification and diagnosis prediction [47-50]. The prediction of interruptions in medical visits requires the use of survival time analysis to build a model. However, there are few studies that have used ML for this purpose. In our study, to avoid the proportional hazard assumption of the Cox regression model and learning difficulties because of imbalanced data, we implemented a ranking method and showed that the scores calculated for each patient using the parameters obtained from the training data were useful for predicting TD, as shown in Table 3.

Our method is a novel way of constructing a survival regression model, and our experimental evaluation showed that it outperformed the existing Cox model in terms of the C-index and AUROC and AUPRC measures and that it would be a useful option for imbalanced data such as TD. The obtained level of performance was not significantly superior to that of the Cox regression model with regard to CIs. Nonetheless, it was not inferior. Many prediction tasks in the clinical domain require that imbalanced data be addressed by prediction models using survival time analysis. Our modeling method does not require the proportional hazard assumption of the Cox regression model and avoids the problem of learning from imbalanced data. It has no variable assumptions, which allowed us to use approximately 150,000 features. Therefore, we believe that our method is a new option for survival regression models in the clinical field.

### Limitations

Our study had several important limitations that must be mentioned. First, the data were obtained from just one hospital. In addition, the test data were obtained by splitting up the data from just one hospital. They may not be entirely representative of other regions because of the different implementations and degrees of diabetes care. Consequently, the results of this study are not sufficient to assess the generalizability of our method; a study using more data from different hospitals will be required.

Second, the participants with a history of TD in this study represented only 1 subgroup of patients. Some could have discontinued treatment temporarily, and we were unable to capture these patients in this study. Moreover, if a patient changed clinics without notice and continued treatment elsewhere without any evidence in the EHR, their case would have been judged as TD cases, even if that would not have been accurate. Nonetheless, because this study relied on EHR information, the findings serve the purpose of evaluating the accuracy of the model using real-world data.

Third, our method used a large number of features and optimized them with the L2-norm regularizer, which made it difficult to find features of high importance that contribute to the prediction. In the future, we intend to investigate ways to improve interpretability, such as by using explainable artificial intelligence and Lasso regularization.

Fourth, a large number of features were generated in the predefined procedure, and the inherent trends and meanings of each feature in itself are not adequately considered. The features need to be designed more appropriately to improve the interpretability of the results.

Fifth, our method was superior to the binary classification model in that it could compare a patient’s risk of TD with the time remaining until TD. However, it requires O(n^2^) pairs to learn the model parameters, whereas a binary classification requires only O(n) records for n training data. We need to reduce the computational cost.

Finally, it should be noted that as ML generally reflects the characteristics of the majority, our results suggest that the predictive performance obtained in this study cannot be applied to a minority of clusters in the population, such as pediatric patients.

### Conclusions

We developed a novel prediction model for calculating the TD risk score by applying a machine-learned ranking model to EHR data. This score showed high prediction performance and outperformed the Cox regression model. Our model can alert clinicians about the risk of TD in advance and would be useful in improving patient outcomes by providing room for interventions to avoid interruptions and support diabetes care. In addition to estimating the TD risk score, we are studying ways to predict glycemic control in patients with diabetes to further improve their care.

## Abbreviations

AUPRC: area under the precision-recall curve
AUROC: area under the receiver operating characteristic curve
EHR: electronic health record
HbA_1c_: hemoglobin A_1c_
MA: missed appointment
ML: machine learning
NTT: Nippon Telegraph and Telephone Corporation
TC: treatment continuation
TD: treatment discontinuation

## Data Availability

The data in this study are not openly available because of the restrictions imposed by the research ethics committees that approved this study.
